# Type material of *Plasmodium ovale* sensu lato

**DOI:** 10.4102/sajid.v39i1.615

**Published:** 2024-03-29

**Authors:** Miles B. Markus

**Affiliations:** 1 Wits Research Institute for Malaria, Faculty of Health Sciences, University of the Witwatersrand, Johannesburg, South Africa; 2 School of Animal, Plant and Environmental Sciences, Faculty of Science, University of the Witwatersrand, Johannesburg, South Africa

## Background

After the malaria parasite *Plasmodium ovale* sensu lato (s.l.) had been found to be two species, new scientific names were proposed.^1^ This was not done as stipulated in the International Code of Zoological Nomenclature (ICZN),^2^ thereby making the names invalid according to the Code. The situation is reminiscent of a similar one that arose half a century ago regarding some related apicomplexan protozoa.^3^ Scientists have been talking, mainly informally, about how this can be rectified for *P. ovale* s.l. In accordance with established practice, deposition of type blood slides of both species in a museum, ideally in East Africa,^1^ would normally be required. However, the two species concerned are morphologically indistinguishable,^1,4^ and a case has accordingly been made for there not to be any designated type slides.^4^ A second reason given for the undesirability of going the usual route^1,2^ is related to the widespread historical use of the two scientific names in question,^1,4^ over a long period.^4^ To be able to refer to these significantly pathogenic organisms^5,6,7^ intelligibly by name is a matter of global malariological importance. Also, it is uncertain which of the two species was originally described as *P. ovale*.^4^

To try to resolve this issue in an alternative way to that which has been outlined by Šlapeta et al.,^1^ it would be necessary to apply to the International Commission on Zoological Nomenclature for a ruling. The application, in a prescribed format, would be published in the Bulletin of Zoological Nomenclature as a ‘Case’ and the ruling, after giving the community of malariologists at large (and others) an opportunity to comment, as an ‘Opinion’.

## Discussion

To consider what the conventional procedure should normally be in theory, it would in this instance be better (the parasite being important^5,6,7^) not to put all our eggs in one basket but rather to deposit type blood slides (despite the fact that they would probably be useless practically^1,4^) in museums in at least three countries. The museums should not all be on the same continent, but one of them should certainly be in the country of origin of the material, as correctly suggested.^1^ This would mean lodging the holotype slide (for the species that is to be newly named) and the neotype slide (for *P. ovale* sensu stricto; what that is, would be a calculated decision made in accordance with the ICZN^2^) in one museum and paratype and paraneotype slides in the others. Obviously, the blood smears for each of the two species must be from the same patient. It might have to be assumed that the associated genetic analyses^1^ will have excluded the possibility of mixed infections, which can have a problematic prevalence and could easily be overlooked.^8^ Mosquito-based double-checking^8^ would be desirable. The necessary inclusion of genotypic details^1,4^ illustrates how the selection of type material information for parasitic protozoa such as *Plasmodium* can be less straightforward than for most other animals.^2,9^

It is relevant to observe that the wording ‘the two species’ above refers to two species of parasite, not to two species of malaria. Even though ‘species of malaria’ frequently appears in the literature, there is no such thing. ‘Malaria’ is the name of the disease, not the causative organism.^10^ Thus, the expression ‘species of malaria’ is nonsensical. This remarkably ubiquitous error is something to be avoided in future publications on *P. ovale* s.l., taxonomic or otherwise.

Also on the subject of terminology, the correct way to refer to past findings reported in publications on ‘*P. ovale*’ that could have been for either of the species or both (mixed infection) is to use the appellation ‘*Plasmodium ovale* sensu lato’. Likewise, this terminology (which appears in the ICZN^2^) should be used in relation to current and future work when genetic identification has not been carried out.

Although the term ‘paraneotype’ is not covered by the ICZN,^2^ it is nevertheless being widely used, unofficially, in zoology. More specifically, the use of ‘paraneotype’ in conjunction with ‘neotype’ has authoritatively been recognised for haemosporidian parasites,^11^ into which group *P. ovale* s.l. falls. In the event that the established taxonomic practice described here were eventually to be followed, it would be in order to use the word ‘paraneotype’ when assigning type slide material (for the designated original *P. ovale* s.l. taxon) that is additional to the single neotype slide.

## Conclusion

At this point, the International Commission on Zoological Nomenclature could be approached, as explained here, about the complicated *P. ovale* s.l. nomenclatorial situation.
